# Cryoballoon ablation with left lateral decubitus position in atrial fibrillation patient where the left atrium was compressed by the vertebra

**DOI:** 10.1002/ccr3.1069

**Published:** 2017-07-12

**Authors:** Yosuke Nakatani, Yoshiaki Yamaguchi, Tamotsu Sakamoto, Koichiro Kinugawa

**Affiliations:** ^1^ Second Department of Internal Medicine University of Toyama Toyama Japan

**Keywords:** Atrial fibrillation, catheter ablation, left lateral decubitus position

## Abstract

Catheter ablation of atrial fibrillation is difficult when the left atrium is compressed by the vertebra. The heart may shift forward, and compression of the left atrium may be relieved in the left lateral decubitus position. Therefore, catheter ablation could be performed in the left lateral decubitus position even in such cases.

## Introduction

Catheter ablation (ABL) has become a commonly practiced therapy for atrial fibrillation (AF), especially against drug resistant AF. However, compression of the left atrium by the aorta and the vertebra is not uncommon 1. In such cases, it is difficult to perform ABL because the risk of cardiac perforation during transseptal puncture is increased and catheter maneuverability is limited.

## Case Presentation

**Figure 1:**
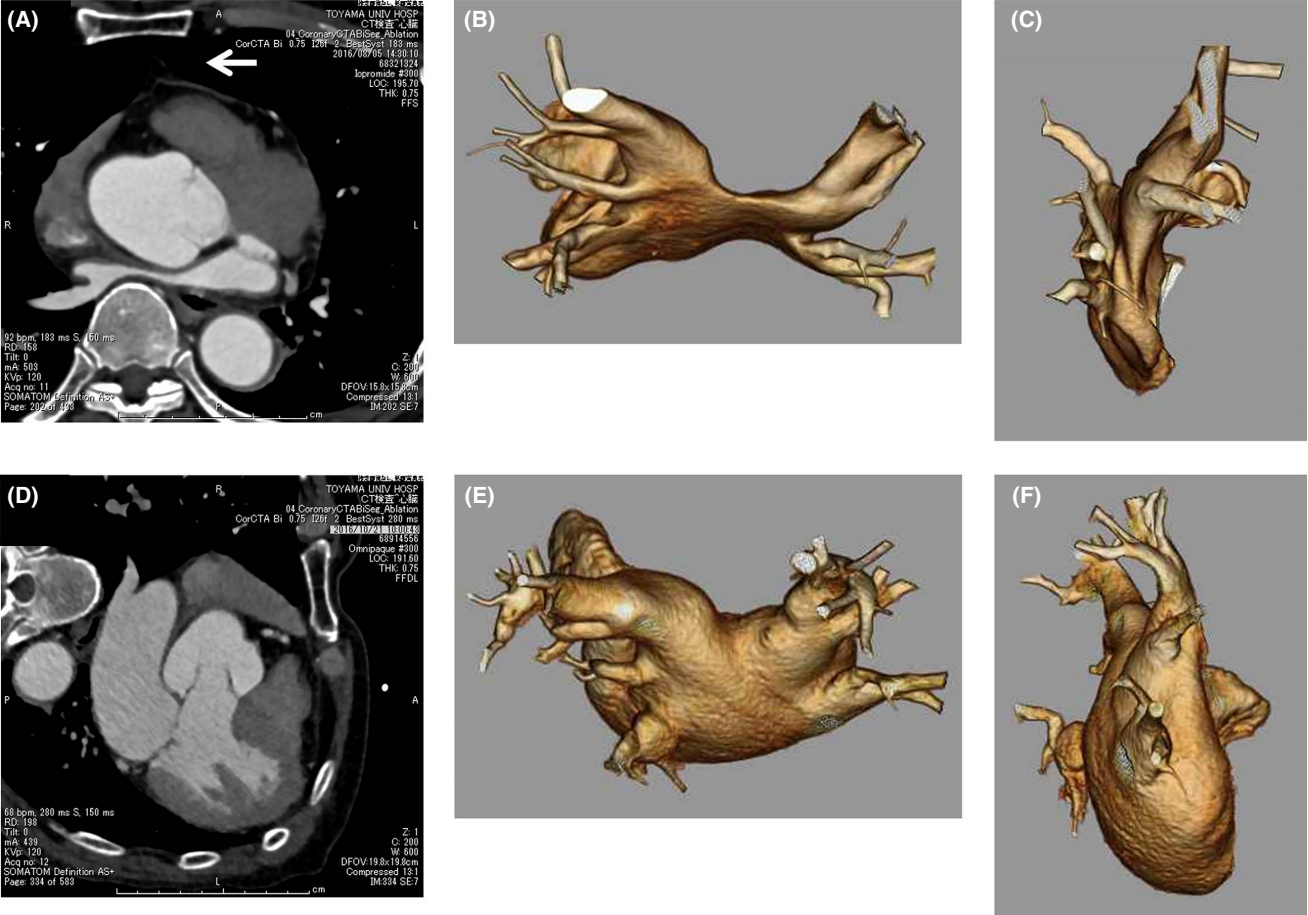
Enhanced chest computed tomography in supine position (A, B, C) and left lateral decubitus position (D, E, F). (A) There was a space in front of the heart (arrows) because of backward shift of the heart in supine position. Left atrium was compressed by mildly enlarged aortic root (maximum aortic root diameter was 41 mm) and the vertebra. Superior view (B) and right lateral view (C) of the left atrium showed extremely small left atrial dimension. (D) Compression of left atrium was relieved because of frontward shift of the heart in left lateral decubitus position. The space in front of the heart disappeared. Superior view (E) and right lateral view (F) of the left atrium showed normal left atrial dimension.

A 67‐year‐old man was referred for ABL of AF. However, enhanced computed tomography (CT) performed before ABL revealed that the left atrium was compressed by mildly enlarged aortic root and the vertebra (Fig. 1A) and that the left atrial dimension was too small to manipulate the catheters (Fig. 1B and C). ABL was canceled, and pharmacotherapy with cibenzoline and verapamil was started; however, AF frequently occurred in spite of the pharmacotherapy. It was suggested that the heart shifted backward, because wide space was observed in front of the heart. Therefore, enhanced CT was performed in the left lateral decubitus position (Fig. 1D–F). As a result, the heart shifted forward and compression of the left atrium was relieved.

**Figure 2:**
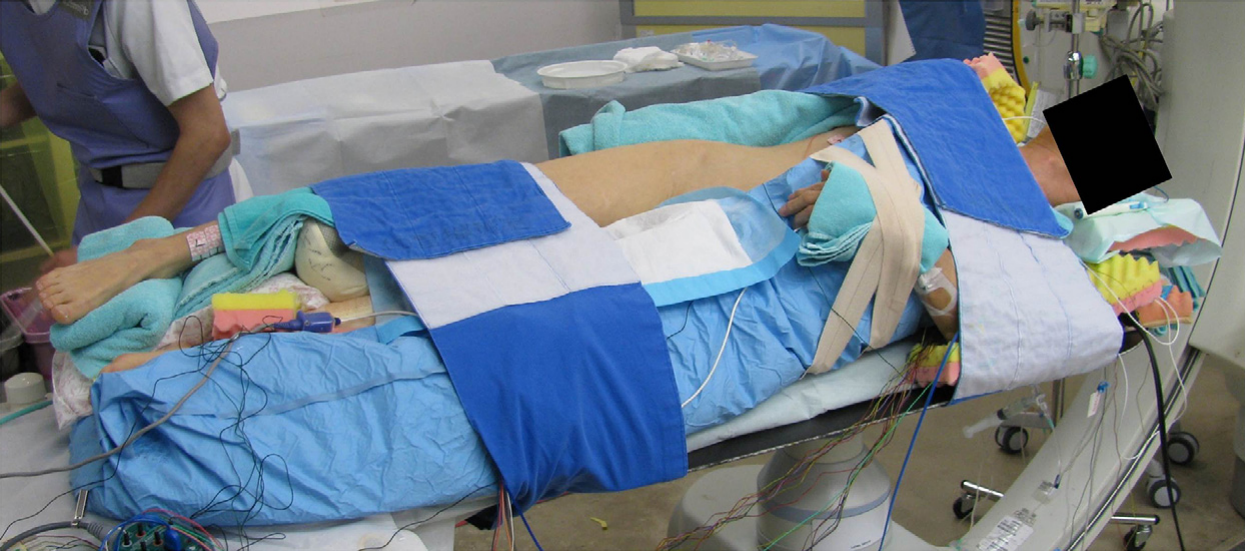
Left lateral decubitus position during catheter ablation. The patient was fixed in the left lateral decubitus position at approximately 45° with a patient immobilization system. Cushions were placed around the left arm to prevent axillary nerve palsy.

**Figure 3:**
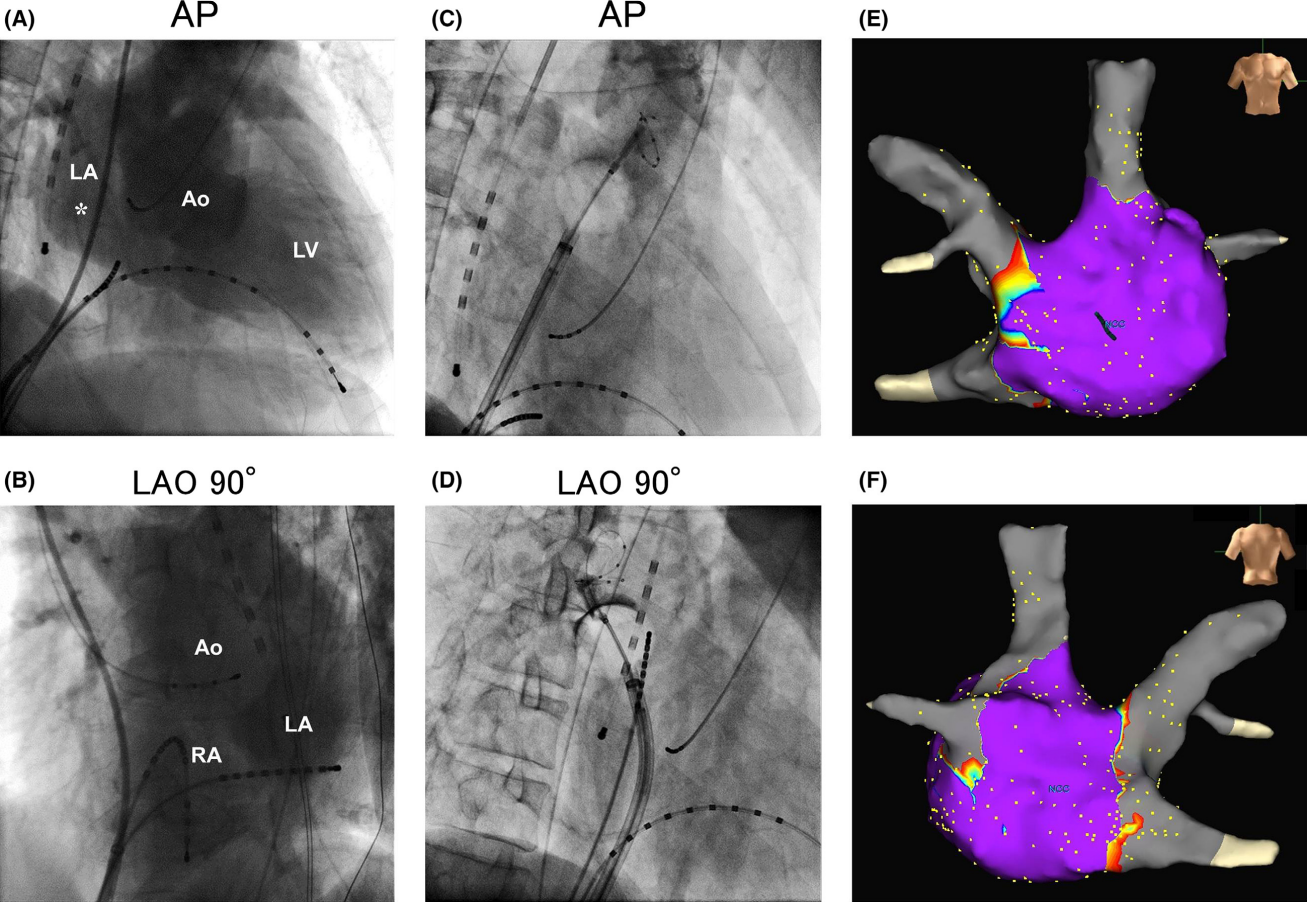
Right atrial angiography (A, B), fluoroscopic image of the cryoballoon ablation (C, D), and voltage map after the cryoballoon ablation (E, F). Anterior–posterior (AP) view (A) and 90° left anterior oblique (LAO) view (B) of the delayed image of the right atrial angiography. There was enough space in the left atrium to perform transseptal puncture. Puncture site is indicated by asterisk. Fluoroscopic image of the cryoballoon ablation to the left superior pulmonary vein (C) and the right superior pulmonary vein (D). The cryoballoon was inflated without any problems in the left atrium. Voltage map created after the ablation (E, F) revealed that the antrum of the pulmonary vein was widely ablated. Ao, aorta; LA, left atrium: LV, left ventricle; RA, right atrium.

ABL was performed in the left lateral decubitus position. Taking into account the range of the fluoroscopic projection angle, the patient was fixed in the left lateral decubitus position of approximately 45° using a patient immobilization system (ESFORM, Engineering System Co, Ltd, Nagano, Japan) (Fig. 2). Electrode catheters were inserted into the right femoral vein and the right femoral artery after fixing in the left lateral decubitus position, because fixing during clean operation was difficult. ABL was performed in a posterior–anterior view and a 90° left anterior oblique (LAO) view (ABL is usually performed in a 35° right anterior oblique view and a 45° LAO view at our institution). Right atrial angiography revealed that there was enough space to perform a transseptal puncture (Fig. 3A and B). The transseptal puncture was performed using a 8‐Fr SL0 sheath (St Jude Medical, Inc, St Paul, MN) and a radiofrequency needle (Japan Lifeline Co., Ltd, Tokyo, Japan). Pulmonary vein angiography was performed, and the three‐dimensional geometry of the left atrium was created on the NavX system (St Jude Medical, Inc) before ABL. Pulmonary vein isolation was successfully performed with a 28‐mm Arctic Front Advance cryoballoon (Medtronic, Inc, Minneapolis, MN) (Fig. 3C and D). A voltage map performed after ABL revealed that the antrum of the pulmonary vein was successfully ablated (Fig. 3E and F). The patient was discharged without any complications, and thereafter, no AF recurrence was observed.

## Discussion

Transseptal puncture is necessary for the approach to the left atrium, but there is a risk of cardiac perforation by puncturing the left atrial wall if the left atrium does not have enough space 2. The risk of cardiac perforation can be reduced using a radiofrequency needle 3 and intracardiac echocardiography 4, but the risk of cardiac perforation is not completely eliminated in cases where the left atrium is extremely narrow. In the present case, the left atrium was compressed by mildly enlarged aortic root and the vertebra in the supine position, and it was difficult to perform a transseptal puncture and manipulate the catheters in the left atrium.

In the present case, we suspected that the heart was shifted backward during supine position because there was a space in front of the heart (Fig. 1A). Although the heart is anchored at its base by relatively rigid tissue, the apical end of the heart can significantly shift position due to movement of the patient 5. A previous study using transesophageal echocardiography revealed that changing the body position from supine to lateral causes a shift in the position of the heart 6. In the present case, the heart shifted forward in the left lateral decubitus position and compression of the left atrium was relieved. Although the frequency is unknown, similar cases may be included in cases where the left atrium is compressed by the aortic root and the vertebra. Therefore, it may be worth performing enhanced CT in the left lateral decubitus position in such cases.

The most known cause for a large shift of the heart is congenital defects of the pericardium 7. Coexistence of congenital defects of the pericardium was a concern in the present case. There is a risk of massive intrapleural hemorrhage if cardiac perforation occurs in patients with congenital defects of the pericardium. In addition, it was difficult to precisely manipulate a radiofrequency ablation catheter due to the difference in direction of fluoroscopy in the present case. A previous study reported that a pericardial tamponade was seldom caused by cryoballoon ablation 8. Accordingly, the cryoballoon catheter was chosen for ABL rather than a radiofrequency ablation catheter. Pulmonary vein angiography and creation of the three‐dimensional geometry were performed before ABL to confirm there was enough space for using the cryoballoon catheter in the left atrium.

In the present case, cardiac magnetic resonance imaging was evaluated after ABL; however, defects of the pericardium were not observed. It seems that the fixation of the heart by the supporting tissue was loose in the present case. There were no clinical characteristics suggesting Marfan syndrome or Ehlers–Danlos syndrome except for mild enlargement of the aortic root in the present case. It is important to recognize that the position of the heart can largely shift even in patients without comorbidities such as defects of the pericardium.

Straight back syndrome is a thoracic deformity with absence of upper thoracic spine kyphosis 9. It reduces anteroposterior diameter of the chest and causes compression of the left atrium by the vertebra. However, straight back syndrome was unlikely in the present case because anteroposterior diameter of the chest was normal.

The present case suggests that ABL could be performed in the left lateral decubitus position even in some patients where the left atrium is compressed by aortic root and the vertebra.

## Conflict of Interest

None declared.

## Authorship

YN: contributed to concept/design and drafted the article. YY, TS, and KK: made critical revision of article.
